# Reference Genome Choice Shapes RNA-Seq Quantification and Downstream Interpretation in Chinese Breast Cancer Patients

**DOI:** 10.3390/biomedicines14102296

**Published:** 2026-10-09

**Authors:** Ruiyang Zhang, Yuan Peng, Mengmeng Zhang, Yanan Chu, Changjun Shao, Zhuo Huang, Yiji Yang, Jing Chen, Yu Kang, Shu Wang

**Affiliations:** 1 China National Center for Bioinformation, Beijing Institute of Genomics, Chinese Academy of Sciences, Beijing 100101, China; zhangruiyang@big.ac.cn (R.Z.); chuyn@big.ac.cn (Y.C.); shaochj@big.ac.cn (C.S.); huangzhuo@big.ac.cn (Z.H.); yangyiji@big.ac.cn (Y.Y.); chenj@big.ac.cn (J.C.); 2 Beijing Institute of Genomics, University of Chinese Academy of Sciences, Beijing 100049, China; 3 Breast Center, Peking University People’s Hospital, Beijing 100044, China; 13671287670@163.com (Y.P.); zhangmm428@163.com (M.Z.)

**Keywords:** population-matched reference genome, telomere-to-telomere genome, T2T-YAO, breast cancer, RNA-seq, transcriptomic inference, biomarker discovery, single-cell RNA-seq

## Abstract

**Background/Objectives**: Reference genome choice can introduce systematic bias into cancer transcriptomic analyses, particularly for populations underrepresented in standard references. However, its impact on downstream biological interpretation remains insufficiently characterized. **Methods**: We compared GRCh38, T2T-CHM13, and the East Asian-matched T2T-YAO using bulk RNA-seq data from 148 Chinese breast cancer patients and single-cell RNA-seq data comprising 65,968 cells from paired primary tumors and lymph node metastases. We assessed reference-dependent differences in read mapping, gene expression, and downstream transcriptomic analyses. **Results**: Compared with GRCh38, YAO reduced multi-mapped reads approximately three-fold and increased confidently exon-assigned reads. Re-mapping YAO-assigned reads to GRCh38 showed that 19.91% changed gene assignment, with a mean of 1016 genes per sample exhibiting more than ten-fold expression differences. Across the cohort, 8432 genes showed such discrepancies, and 68.49% overlapped low-mappability regions in YAO. These genes were enriched in receptor, ion channel, and drug metabolism functions. Reference-dependent expression differences propagated to differential expression, immune cell inference, fusion detection, and classifier performance. In single-cell analyses, T2T references retained more cells after quality filtering and resolved a fibroblast subpopulation missed by GRCh38. **Conclusions**: Reference genome selection substantially influences RNA-seq quantification and downstream biological interpretation in breast cancer. Population-matched and complete T2T reference genomes can reduce mapping ambiguity and improve the resolution of transcriptomic features, highlighting reference genome choice as an important and underappreciated source of systematic bias in cancer transcriptomics.

## 1. Introduction

The human reference genome provides the coordinate framework for virtually all omics analyses, including transcriptomics. However, GRCh38—the long-standing but incomplete reference—introduces biases due to residual assembly gaps and its mosaic composition from individuals of mixed ancestry [1,2]. Recent advances in long-read sequencing have enabled the generation of gapless telomere-to-telomere (T2T) assemblies, such as T2T-CHM13 (hereafter CHM13), which substantially improve small and structural variant detection [3,4] and affect transcriptome analysis [5]. Yet its European origin limits its suitability for studies involving other populations. Given the genetic diversity across human populations, relying on a single reference genome creates a systematic bias [6,7,8], precisely the issue that ancestry-matched references aim to mitigate. The recently assembled T2T-YAO (hereafter YAO), a complete reference genome derived from an East Asian individual, offers a population-matched framework for genomic and transcriptomic studies in Chinese cohorts [9,10,11]. Our previous exon analysis indicated that YAO can reduce the detection rate of benign variations and improve the accuracy of variant identification [10]. However, how the use of reference genomes from different ancestral origins influences transcriptome analyses remains unclear.

Transcriptome analysis is inherently more complex than variant calling, as it depends not only on accurate read alignment and annotation but also on the precise quantification of gene expression, which underpins downstream biological interpretation and feature selection for disease stratification [12,13]. Expression profiles further serve as the basis for cell type annotation in single-cell studies and for elucidating pathogenic mechanisms at cellular resolution [14,15]. A recent study reported that the use of the CHM13 reference influenced the quantification of approximately 40% of human genes [5], underscoring the potential impact of reference genome choice. However, how reference-related misalignment affects gene expression quantification at both the bulk and single-cell levels—and how these differences propagate to downstream biological discoveries—remains largely unexplored.

Here, we systematically compared the performance of GRCh38, CHM13, and YAO using bulk RNA-seq data from a Chinese breast cancer cohort comprising patients with early lymph node metastasis (LN+, representing an unfavorable prognostic phenotype) and patients without lymph node metastasis (LN−, representing a more favorable prognostic phenotype), together with an independent published single-cell RNA-seq dataset of paired primary tumors and lymph node metastases. We evaluated their impact on mapping accuracy, the identification of differentially expressed genes (DEGs), immune cell composition, and the detection of fusion transcripts, with a particular focus on how these technical differences translate into biologically informative patterns and clinically relevant discrimination between LN+ and LN− patients. Our research demonstrated that the choice of reference genome has a systematic impact on the comparison results and further affects the estimation of gene expression and downstream analysis. T2T references showed broadly similar advantages over GRCh38, while YAO provided a population-matched option for the Chinese cohort. These findings consider the reference genome variations an important source of bias in cancer transcriptome analysis, further highlighting the clinical significance of incorporating its assessment in result interpretation and marker development.

## 2. Materials and Methods

### 2.1. Study Cohort and Samples

The samples for this study were obtained from Peking University People’s Hospital and included a total of 148 Chinese Han patients diagnosed with luminal A breast cancer between 2013 and 2025 (GSA-Human: HRA014762). All tumor tissue samples were preserved by paraffin embedding. The LN+ group comprised patients with small tumors (T1 stage, AJCC) and that had ≥1 metastatic LNs, whereas the LN− group included patients with larger tumors (T2–T3 stages) but no LN metastases (Table S1). To construct the predictive model, the samples were randomly divided into a training set and a validation set. The training cohort comprised 60% cases (55 LN+ and 37 LN−), while the held-out validation cohort included 40% cases (35 LN+ and 21 LN−). The study protocol was approved by the Ethics Committee of Peking University People’s Hospital (Decision No. 2020PHB023-01 of Ethics Committee).

Single-cell RNA sequencing data were obtained from a published study [16] conducted by Fudan University Shanghai Cancer Center. The dataset comprises a total of 65,968 cells from four paired fresh surgical specimens of primary tumors and matched lymph node metastases from four patients, generated using the 10x Genomics single-cell platform.

### 2.2. Bulk RNA Library Construction and Sequencing

For library preparation, we followed a standardized RNA-seq library construction workflow [17]. Total RNA was extracted from FFPE tissue samples using the RNeasy FFPE Kit (QIAGEN, Hilden, Germany). After quantification with the Qubit^®^ RNA HS Assay Kit (Thermo Fisher Scientific, Waltham, MA, USA) and integrity assessment using an Agilent Bioanalyzer (Agilent Technologies, Santa Clara, CA, USA), 50 ng of total RNA was used for library preparation with the Watchmaker RNA Library Prep Kit (Watchmaker Genomics, Boulder, CO, USA), with ribosomal RNA depletion and cDNA synthesis performed according to the integrated workflow. Final library concentration and size distribution were verified using the Qubit 1X dsDNA HS Assay Kit (Thermo Fisher Scientific, Waltham, MA, USA) and an Agilent Bioanalyzer/Qsep system (BiOptic Inc., New Taipei City, Taiwan, China), respectively. Equimolar-pooled libraries were sequenced on the DNBSEQ-T7 platform to acquire about 8 Gb of raw 100 bp PE reads per sample.

### 2.3. Bulk RNA-Seq Data Analysis

For bulk RNA-seq data, the quality of raw sequencing reads was assessed using FastQC (v0.11.5), followed by adapter trimming and quality filtering using TrimGalore (v0.6.10). The filtered clean reads were then in parallel aligned to three reference genomes—GRCh38.p14, CHM13, and YAO—using STAR (v2.7.11b) [18]. The reference genomes and annotations were obtained as follows: GRCh38.p14 and its annotation were obtained from GENCODE (https://www.gencodegenes.org/human/ (accessed on 13 September 2026)), CHM13v2.0 was obtained from GitHub (https://github.com/marbl/CHM13 (accessed on 13 September 2026)), and YAO was derived from the annotations of CHM13 in GENCODE by Liftoff [19] (https://github.com/KANGYUlab/T2T-YAO-resources (accessed on 13 September 2026)). Correspondence between Ensembl genes (ENSG IDs) in GRCh38 and genes annotated in the CHM13 and YAO reference genomes was established by extracting information directly from the annotation files.

Following read alignment, featureCounts (v2.0.8) [20] was employed to count the reads that were uniquely located in the exon regions, thereby obtaining the expression matrix for each gene. The expression level of each protein and long non-coding RNA (lncRNA) gene was normalized to Transcripts Per Million (TPM) values [21].

### 2.4. Assessment of Read Mapping Performance

The proportion of uniquely mapped reads, the mismatch rate per base, and the percentage of reads mapped to multiple loci were obtained from STAR alignment statistics (Log.final.out). High-quality uniquely aligned paired-end reads were defined as reads marked with a unique alignment flag (NH:i:1) and classified as properly paired in STAR-generated BAM files and were quantified for downstream analyses. In addition, featureCounts alignment efficiency was assessed by extracting the proportion of reads successfully assigned to annotated gene features from the featureCounts summary files. Finally, reads categorized as Unassigned_Ambiguity—defined as uniquely mapped reads whose paired ends overlapped different gene annotations—were quantified based on gene annotation information.

### 2.5. Discrepancies of Reads in Aligning to Different References

To directly assess reference-dependent read assignment, reads uniquely mapped to YAO were realigned to GRCh38 while retaining read identities, allowing us to determine whether the same reads remained assigned to the corresponding gene, became multi-mapped, were reassigned to a different gene or other region, or failed to align. Clean reads from training cohort samples that uniquely mapped to protein-coding genes or lncRNAs in YAO were subsequently re-mapped to GRCh38 to evaluate consistency. Read pairs were categorized according to their alignment and gene annotation concordance between YAO and GRCh38. Read pairs were classified as consistent if both ends mapped to the same gene in YAO and GRCh38 or were classified as (1) multi-mapped (aligned to multiple loci in GRCh38), (2) unmapped (failed to align to GRCh38), (3) no-overlap (mapped but without overlapping annotated gene regions in GRCh38), or (4) no-annotation (had at least one end lacking gene annotation in GRCh38).

### 2.6. GO and KEGG Enrichment Analyses

Gene Ontology (GO) enrichment analysis was performed using the clusterProfiler R package with the human gene annotation database org.Hs.eg.db. Enrichment analyses were conducted for the three major GO categories, including Biological Process (BP), Cellular Component (CC), and Molecular Function (MF). Kyoto Encyclopedia of Genes and Genomes (KEGG) pathway enrichment analysis was performed using clusterProfiler with the human KEGG pathway database. For both GO and KEGG analyses, statistical significance was assessed using an adjusted *p*-value < 0.05. Enriched GO terms and KEGG pathways were visualized using bubble plots, with the gene count represented by the *x*-axis and bubble size and *p*-values represented by the color gradient. OR-related terms were excluded from the displayed enrichment results, and the reported GO and KEGG categories therefore represent non-OR-associated enrichments. Their exceptionally high inter-individual genetic and structural variability may drive enrichment artifacts unrelated to reference genome choice [22,23].

### 2.7. Differential Expression Analysis

Differential expression analysis was restricted to protein-coding genes and lncRNAs in the training cohort samples to focus on well-annotated transcript classes with direct relevance to gene-level transcriptomic interpretation and to maintain comparability across reference annotations. DEGs between LN+ and LN- samples were identified using DESeq2 (v1.42.1) [24] and Vtwins [25]. DESeq2 analysis was performed on raw count matrices generated by featureCounts, using a negative binomial model; genes with an adjusted *p*-value < 0.05 and an absolute log2 fold change ≥ 1 were considered significant. Vtwins was adapted for RNA-seq data and applied to paired-sample comparisons, with genes showing an adjusted *p*-value < 0.01 defined as significant.

### 2.8. Random Forest Modeling

Random forest models were constructed using the DEGs identified by DESeq2 and Vtwins in the training cohort. Model implementation was performed in R (v4.3.1) using the randomForest [26] and caret [27] packages [28]. Samples in the training cohort were used to construct the random forest model. Candidate feature subsets were first evaluated using repeated five-fold cross-validation within the training cohort. The feature subset yielding the optimal cross-validation performance was selected for subsequent model construction. Feature importance was then estimated using the Mean Decrease Gini criterion from a random forest trained on the selected features in the complete training cohort. Model discrimination was assessed by the area under the receiver operating characteristic curve (AUC-ROC), which summarizes the ability of the classifier to distinguish LN+ from LN− samples across classification thresholds. The finalized model was subsequently evaluated in the independent held-out validation cohort, which was not used for feature selection or model optimization. AUC-ROC values were reported together with the 95% confidence interval.

### 2.9. Immune Infiltration Analysis

Immune cell composition was inferred using CIBERSORT [29,30] on TPM-normalized expression profiles in all samples. For each sample, the relative abundances of 22 immune cell types were independently estimated using transcriptomes derived from the GRCh38, YAO, and CHM13 reference genomes. Differences in immune cell proportions between LN+ and LN− groups were evaluated separately for each reference genome, with statistical significance defined as an adjusted *p*-value < 0.05. The pairwise similarity of immune cell composition profiles across reference genomes was assessed using Pearson correlation coefficients to quantify reference-dependent consistency in CIBERSORT-derived estimates.

### 2.10. Fusion Transcript Analysis

Fusion transcript detection was performed using STAR-Fusion (v1.14.0) [31,32] in the training cohort (*n* = 92). For GRCh38 and CHM13, analyses were conducted using the corresponding pre-built reference databases, GRCh38_gencode_v44_CTAT_lib and T2T-CHM13_CTAT_lib [33], respectively. For the YAO reference genome, a customized fusion detection database was constructed according to a previously described method [32] using the YAO reference FASTA and GTF files, a fusion gene annotation library (fusion_annot_lib.gz), protein domain annotations from Pfam-A.hmm [34] and a curated database of human transposable elements from Dfam [35]. Fusion detection was performed using STAR-Fusion default filtering parameters, followed by systematic aggregation and analysis of fusion events across samples.

### 2.11. Single-Cell RNA-Seq Analysis

Single-cell RNA sequencing data were processed using the Cell Ranger pipeline for read alignment, gene quantification, and initial quality control. Downstream analyses were conducted using Seurat (v4.4.0) [16]. Cells were retained for analysis if they met the following quality criteria: (i) total UMI counts and the number of detected genes within ±2 standard deviations of their respective means and (ii) a mitochondrial gene content <10%, calculated using the PercentageFeatureSet function (pattern = “^MT-”). The filtered expression matrix was normalized, and the top 10,000 highly variable genes (HVGs) were identified. Dimensionality reduction was performed using principal component analysis (PCA), and the top 20 principal components were used for cell clustering at a resolution of 0.4, followed by Uniform Manifold Approximation and Projection (UMAP) visualization. Cell type annotation was performed based on established canonical marker genes and the “Human Primary Cell Atlas” database [36], including epithelial cells (EPCAM, KRT19, PROM1, CDH1, KRT8, KRT18), endothelial cells (PECAM1, CDH5, CD34, VWF), B cells (CD79A, CD79B, CD19, IGHD), T/NK cells (CD3C, CD3G, FCGR3A, NKG7), and fibroblasts (COL1A1, COL1A2, COL3A1, ACTA2) [16].

### 2.12. Clustering Consistency Across Reference Genomes

To evaluate the consistency of the single-cell clustering results across different reference genomes (GRCh38, YAO, and CHM13), multiple quantitative metrics were computed, including cell counts, the Adjusted Rand Index (ARI), Normalized Mutual Information (NMI), PCA average correlation, PCA structural similarity, UMAP average correlation, UMAP structural similarity, and UMAP root mean square error (RMSE) [37].

## 3. Results

### 3.1. A Comparative Evaluation of RNA-Seq Alignment Precision Between GRCh38 and the T2T-YAO Reference Genome

Prior to comparative analyses, and considering that all samples were from female patients, chromosome Y was excluded from all mapping and analyses. To ensure data quality, samples with a total data size greater than 5.5 GB were considered valid for inclusion in subsequent analyses. We then proceeded to ensure comparability across reference genomes; we first standardized gene annotation for YAO. Using LiftOff, we successfully transferred 98.4% (62,481/63,494) of gene annotations from the CHM13 v2.0 primary assembly to YAO. Among the 1013 genes that failed to transfer (1.6%), only 180 were protein-coding. For successfully lifted genes, the mean transcript identity relative to GRCh38 exceeded 0.96, demonstrating high annotation fidelity and establishing a robust, comparable foundation for cross-genome analyses (Figure S1).

We then compared the mapping efficiency of bulk RNA-seq reads from our training cohort to both the GRCh38 reference and YAO using STAR. While the overall mapping rate was statistically similar between the two references, GRCh38 showed a slightly higher mapping rate (65.28% ± 13.49% vs. 64.89% ± 13.41%, *p* = 1.93 × 10^−22^, paired *t*-test), probably due to the marginally higher number of genes in GRCh38 (Figure S2A). However, a detailed examination of alignment precision metrics revealed distinct and significant differences. Alignments to YAO exhibited a consistently lower per-base mismatch rate (0.85% ± 0.16% vs. 0.87% ± 0.16%, *p* = 3.24 × 10^−34^) (Figure S2B), and the proportion of multi-mapped reads was approximately three-fold lower in alignment with YAO than with GRCh38 (3.01% ± 0.66% vs. 9.86% ± 3.19%, *p* = 1.93 × 10^−39^) (Figure S2C).

**Figure 1:**
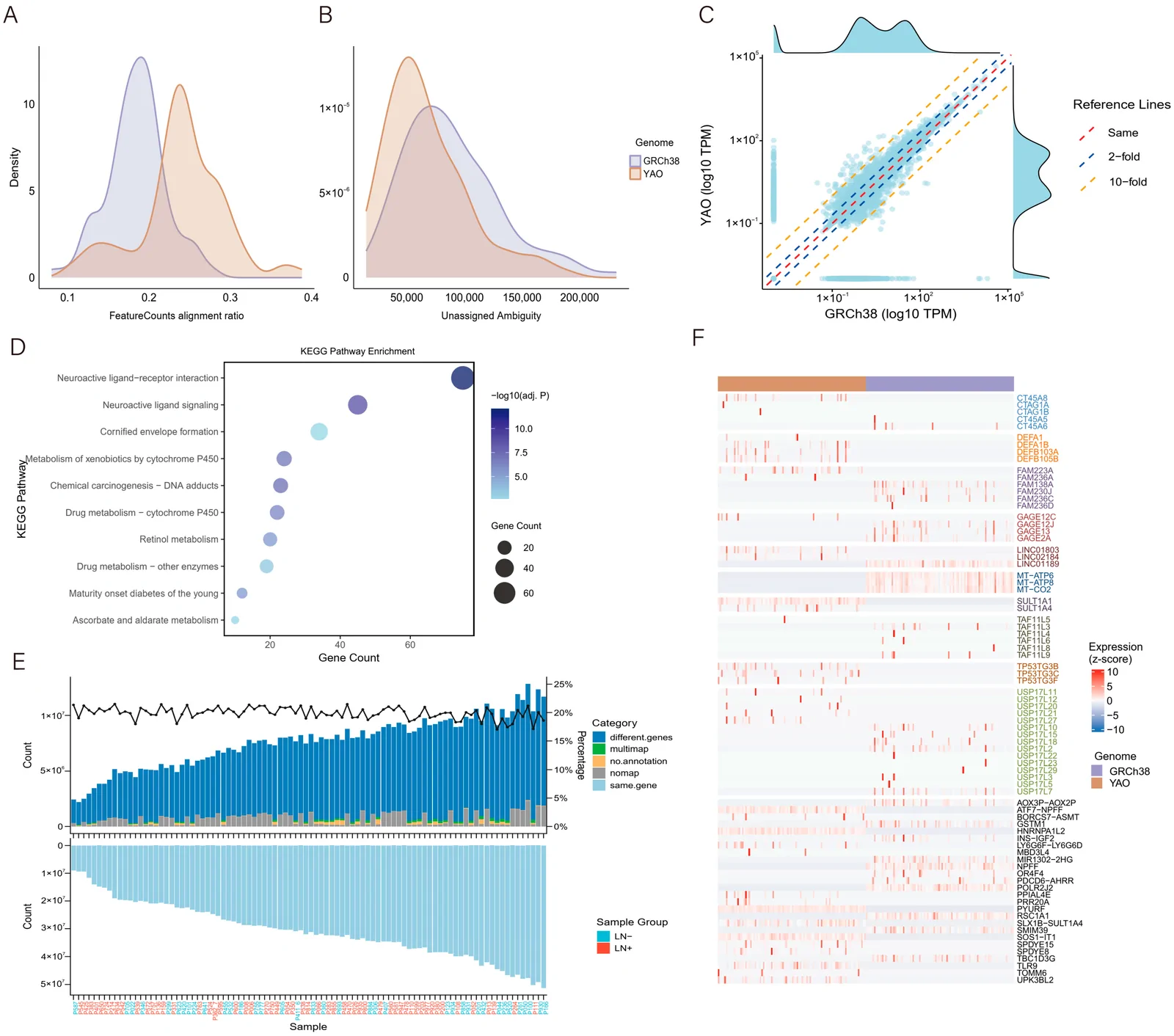
A comparative assessment of RNA-seq alignment and gene expression quantification between the GRCh38 and YAO reference genomes. (**A**). The proportion of successfully aligned reads on exons (alignment ratio) for each sample using the GRCh38 (purple) and YAO (orange) genomes. (**B**). The number of paired reads that are assigned to different genes. (**C**). A scatter plot comparing gene expression levels (TPM) quantified in YAO versus GRCh38 for all genes. The central red line indicates perfect equivalence (x = y). The flanking blue and yellow lines demarcate thresholds for 2-fold and 10-fold expression differences, respectively. (**D**). KEGG enrichment analyses were performed for 8432 genes, showing more than a ten-fold expression difference between YAO and GRCh38. Dot size represents the number of genes, and color indicates the statistical significance of enrichment. (**E**). A re-mapping analysis of reads uniquely aligned to YAO. The stacked bar chart categorizes the genomic fate of these reads when aligned against GRCh38: mapping to the same gene, different genes, multiple loci (multimap), unannotated regions, or failing to align (nomap). The line graph tracks the cumulative proportion difference across these categories between the two genomes. (**F**). The heatmap shows the sets of genes detected as uniquely expressed when using either the GRCh38 or YAO reference.

The enhanced mapping specificity achieved with YAO directly translated to a substantial improvement in gene-level quantification. Using featureCounts to assign uniquely mapped reads to annotated exons, we found that YAO yielded a significantly higher proportion of reads uniquely and confidently assigned to a single gene (23.94% ± 5.35% vs. 18.41% ± 3.58%, *p* = 1.05 × 10^−33^) (Figure 1A). Correspondingly, the number of reads categorized as “Unassigned_Ambiguity”—those overlapping multiple genes and thus unassigned—was reduced by nearly 23.5% when using YAO (68,950.55 ± 35,998.14 vs. 90,232.22 ± 41,629.74, *p* = 1.89 × 10^−31^) (Figure 1B).

Overall, these results indicate that YAO yielded higher read assignment specificity and fewer ambiguous alignments under the present annotation and alignment framework. This improvement likely reflects the increased completeness and population-matched sequence context of YAO, which better resolves homologous and repetitive regions during alignment.

### 3.2. Mapping Results Affect Expression Quantification

To assess the translational impact of mapping specificity on gene expression quantification, we first limited downstream analyses to protein-coding genes and long non-coding RNAs (lncRNAs) to ensure biological relevance and comparability. In a representative sample, reads uniquely mapped to YAO were realigned to GRCh38. This revealed that approximately 19.49% either failed to align or became multi-mapped. A comparison of the TPM-normalized gene expression of the sample inferred from YAO and GRCh38 revealed substantial discrepancies: 2420 genes showed at least a two-fold difference, and among them, 1026 genes exhibited differences exceeding ten-fold (Figure 1C). This pattern was consistent across the cohort, with a mean of 2210 ± 229 genes (6.61%) and 1016 ± 220 genes (3.04%) per sample affected at the two-fold and ten-fold thresholds, respectively. We next extended this analysis across the entire cohort to characterize the broader impact of reference genome choice on gene expression quantification. In total, 8432 non-redundant genes showed more than a ten-fold difference in expression between YAO and GRCh38 across the analyzed samples. Functional enrichment analysis revealed that these genes were predominantly associated with olfactory receptors, neuronal receptors, ion channels, drug metabolism, and other gene families (Figure 1D and Figure S3). Notably, 68.49% (5575 genes) of these genes overlapped regions exhibiting ambiguous read mapping in YAO, suggesting that their pronounced expression differences were largely attributable to differences in read assignment.

To further investigate the basis of these reference-dependent expression changes, we re-mapped all reads uniquely aligned to YAO back onto GRCh38 for every sample. Consistent with the single-sample analysis, approximately 19.91% ± 1.04% of reads were reassigned to different genes, mapped to multiple loci or intronic regions, or failed to align, whereas 80.09% ± 1.04% remained mapped to the same gene in GRCh38 (Figure 1E). To illustrate these reference-dependent changes at the gene level, we examined two representative examples, KCNJ18 and NPIPA2. While GRCh38 contains a single annotated KCNJ18 copy, YAO contains two copies on opposite strands. Read-level inspection revealed that five representative read pairs assigned to the second KCNJ18 copy in YAO were redistributed upon realignment to GRCh38: one pair mapped to KCNJ12, two pairs exhibited discordant alignments between KCNJ12 and KCNJ18, and two pairs were unmapped (Figure S4). Similarly, NPIPA2 belongs to the highly homologous NPIP gene family, which exhibits documented population-associated copy number variation [38]. Of the 17 read pairs assigned to NPIPA2 using YAO, only two mapped to NPIPA1 upon realignment to GRCh38, whereas the remaining 15 were unmapped (Figure S5). Together, these examples illustrate how differences in reference genome representation can alter read placement and gene-level quantification in highly homologous regions.

Beyond changes in expression magnitude, reference genome selection also affected gene detection. After excluding genes that failed to transfer to YAO, we identified 38 YAO-specific and 39 GRCh38-specific expressed genes (Figure 1F). Although these genes were annotated in both reference genomes, they exhibited reference-specific expression patterns associated with differences in read assignment. Functional annotation revealed that many belonged to multi-gene families, including Cancer/Testis Antigens (CTAs), defensins, and FAM genes, whose high sequence similarity may increase their susceptibility to mapping ambiguity. Collectively, these results demonstrate that reference genome selection can affect both expression estimates and gene detection, with particularly pronounced discrepancies in highly homologous genomic regions, underscoring the importance of reference genome representation for reliable RNA-seq quantification and downstream biological interpretation.

### 3.3. Reference Genome Choice Shapes Differential Expression Analysis and Downstream Predictive Modeling

To systematically assess whether discrepancies in expression quantification arising from different reference genomes propagate to downstream biological discovery, we compared differentially expressed genes (DEGs) between LN+ and LN− groups using GRCh38, YAO, and CHM13. Including CHM13 allowed us to evaluate the influence of population-matched versus non-matched references in our predominantly East Asian cohort.

**Figure 2:**
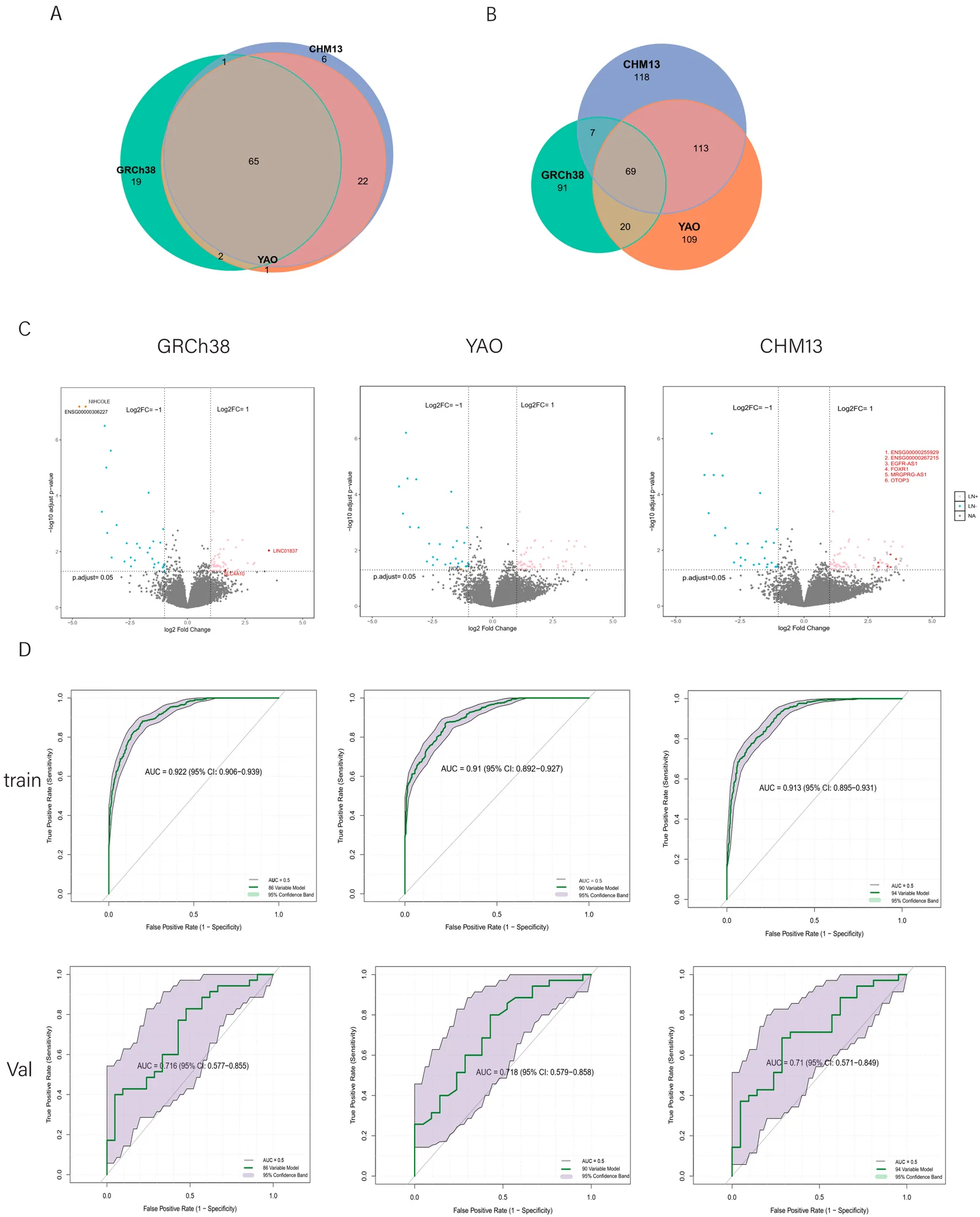
The comparative performance of the GRCh38, YAO, and CHM13 reference genomes in identifying and modeling disease-associated genes. (**A**). DEGs identified between LN+ and LN− groups using DESeq2. The Venn diagram illustrates the overlap of DEG sets called by each reference genome. (**B**). Volcano plots showing differential expression analysis between LN+ and LN− breast cancer samples based on the GRCh38, YAO, and CHM13 reference genomes. The marked DEGs are unique to the reference genome. (**C**). The predictive accuracy of the random forest classifiers. The plot shows the AUC values for both the training and independent validation sets, as modeled using the DESeq2 DEG sets from each reference genome. (**D**). DEGs identified between LN+ and LN− groups using VTwins.

We first applied DESeq2, a widely used count-based differential expression method with built-in normalization. The numbers of DEGs identified by DESeq2 were broadly comparable across the three reference genomes, with 86, 90, and 94 DEGs detected using GRCh38, YAO, and CHM13, respectively. A total of 65 DEGs were shared across all three analyses, whereas each reference genome also yielded a subset of reference-specific DEGs (Figure 2A,B). Notably, among the 18 DEGs uniquely detected using GRCh38, 13 were attributable to GRCh38-specific gene annotations. These findings indicate that reference genome choice can alter the composition of DEG sets even when the overall number of detected DEGs remains similar. To evaluate the biological relevance and predictive power of the DEG sets identified by this approach, we trained random forest (RF) classifiers. Using DESeq2-derived DEGs, models trained on the three reference genomes showed similarly high performance in the training cohort (AUC = 0.910 for YAO, 0.913 for CHM13, and 0.922 for GRCh38). However, performance decreased in the independent validation cohort, with comparable AUC values across references (YAO: 0.718; CHM13: 0.710; GRCh38: 0.716) (Figure 2C). Notably, 89 of the 90 DEGs identified using YAO were shared with at least one of the other two references, and models based on YAO-derived features exhibited greater stability. In contrast, DEGs unique to GRCh38 and CHM13 contributed less to model performance, as indicated by lower feature importance scores (Table S2).

To further identify expression features distinguishing the two groups, we employed Vtwins, a sensitive method for small cohorts with multi-dimensional data conducted by transferring a group cohort to a paired cohort and leveraging within-pair variance to enhance statistical power [25]. Vtwins identified 311, 307, and 186 DEGs using YAO-, CHM13-, and GRCh38-derived expression matrices, respectively. Only 69 DEGs were shared across all three references, but YAO and CHM13 showed greater overlap with 113 genes (Figure 2D). In constructing the random forest classifier, we only used the top 20 Vtwins-derived DEGs from each reference to facilitate fair comparison, where 9 were common across references, and 13 were shared by YAO and CHM13. In the training cohort, the YAO-, CHM13-, and GRCh38-based models achieved cross-validation AUCs of 0.920, 0.880, and 0.878, respectively. In the held-out validation cohort, the corresponding AUCs were 0.754, 0.741, and 0.705, respectively. Overall, the three reference-based models showed broadly comparable predictive performance (Figure S6 and Table S4). These results suggest that the two T2T references provided broadly similar predictive performance, with a modest numerical separation from GRCh38. The YAO-derived classifier also showed a relatively concentrated distribution of feature importance, with KAT6B having the highest score (3.6) and the scores of 17 features ranging from 1.8 to 3.6 (Figure S6). Several highly ranked features, including KDM7A, SMARCD2, and RAD23B, have previously been implicated in cancer-related processes, prognosis, or metastasis [39,40,41]. Overall, these results indicate that reference genome choice can influence feature selection and model performance, while the biological and clinical relevance of individual features requires validation in larger independent cohorts.

### 3.4. Reference Genome Considerations in Single-Cell RNA-Seq Analysis

To assess whether the reference genome-dependent effects observed in bulk RNA-seq extend to single-cell transcriptomic analysis, we revisited the scRNA-seq data of paired primary tumor and metastatic LN samples. Quantitatively, alignment to the T2T references resulted in a consistently higher yield of cells passing quality control filters compared to GRCh38 (tumor: YAO 12,042; CHM13 12,167; GRCh38 11,028; LN: YAO 13,366; CHM13 13,503; GRCh38 11,909).

**Figure 3:**
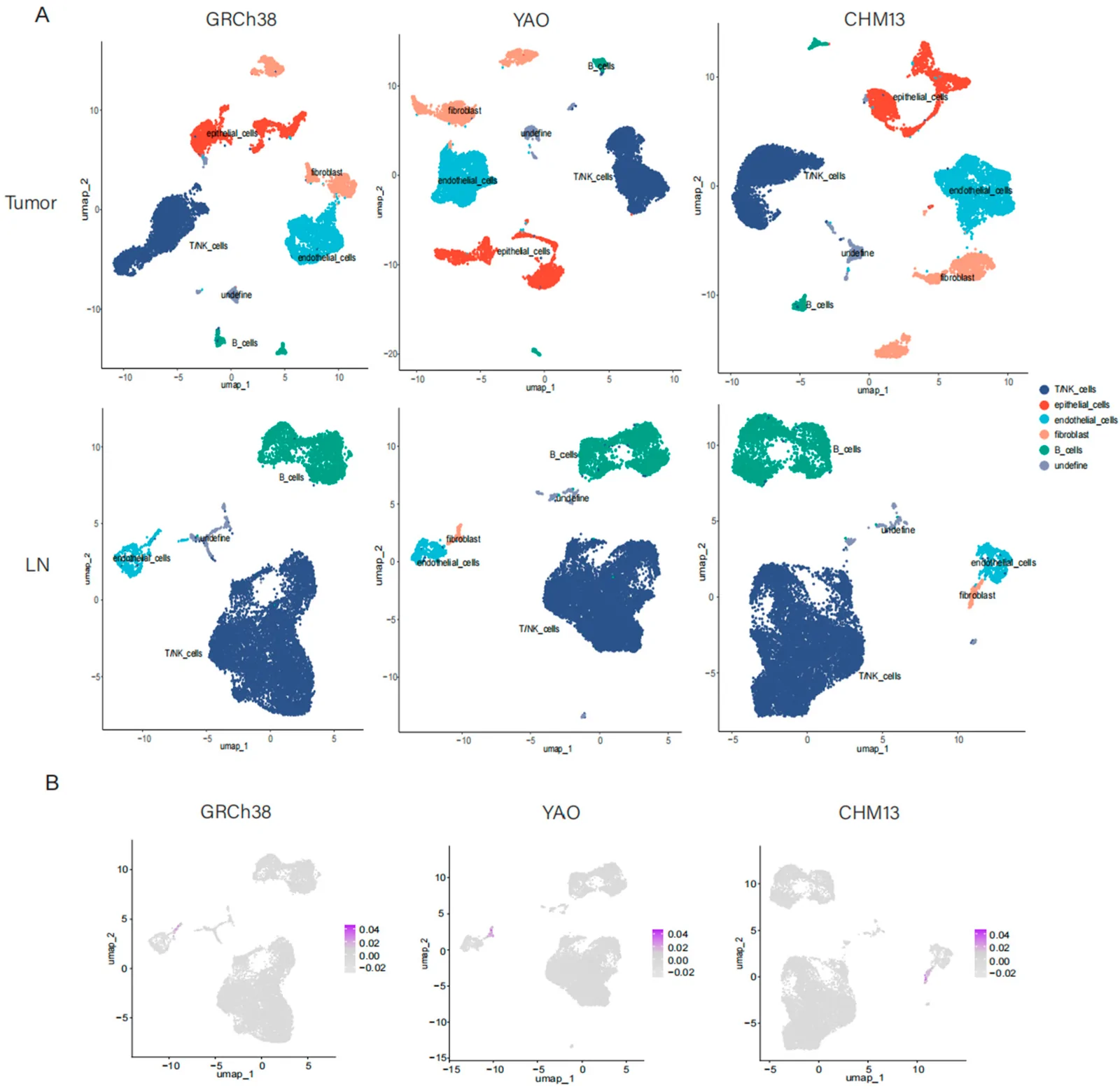
Impact of reference genome selection on single-cell clustering and cell type annotation in paired primary and metastatic tumors. (**A**). UMAP plots showing cell clustering and annotation for single-cell transcriptomes from primary tumor and LN samples, processed independently using GRCh38, YAO, and CHM13 reference genomes. (**B**). Expression levels of fibroblast marker gene COL3A1 visualized on UMAP plots of LN.

Clustering and annotation identified five major cell types across references: T/NK cells, epithelial cells, endothelial cells, fibroblasts, and B cells. While cluster structures in primary tumors were largely consistent, metastatic LN samples revealed a critical, reference-dependent divergence: a discrete fibroblast population was clearly resolved by both T2T references (YAO and CHM13) but was not distinctly identifiable using GRCh38 (Figure 3A). To further corroborate this, we examined the expression level of COL3A1, the well-established fibroblast marker [42]. While it marked a distinct, well-defined cell population in both YAO and CHM13 maps, its expression in the GRCh38-based analysis lacked clear cluster specificity and corresponded with lower per-cell expression levels (Figure 3B and Figure S7). These findings suggest that reference completeness can be critical in detecting small or low-abundance cell populations, in which marker genes are members of a gene family and prone to ambiguous alignment.

To quantify the consistency of cellular landscapes derived from different references, we performed a systematic pairwise comparison. Consistent with the overall trend, cellular mapping and clustering results showed higher concordance between the two T2T assemblies than between either T2T assembly and GRCh38. For instance, in LN samples, the set of cells commonly retained by both YAO and CHM13 was larger (13,344) and exhibited higher clustering similarity (Adjusted Rand Index [ARI] = 0.724; Normalized Mutual Information [NMI] = 0.782) compared to analyses involving GRCh38 (shared cells: 11,690–11,729; ARI = 0.631–0.693; NMI = 0.736–0.744) (Table S4). 

These reference-driven differences in cell population detection directly propagated to downstream molecular characterization. When performing cluster-specific differential expression analysis, the resulting DEG sets identified apparently differed across the three references (Table S5), with 5.29% overlapped in primary tumor and 0.72% overlapped in LN. Thus, the choice of reference genome introduces measurable variability that propagates from fundamental cell type identification to the downstream molecular characterization of those same cell populations.

### 3.5. Reference Genome Effects on Immune Microenvironment Inference

**Figure 4:**
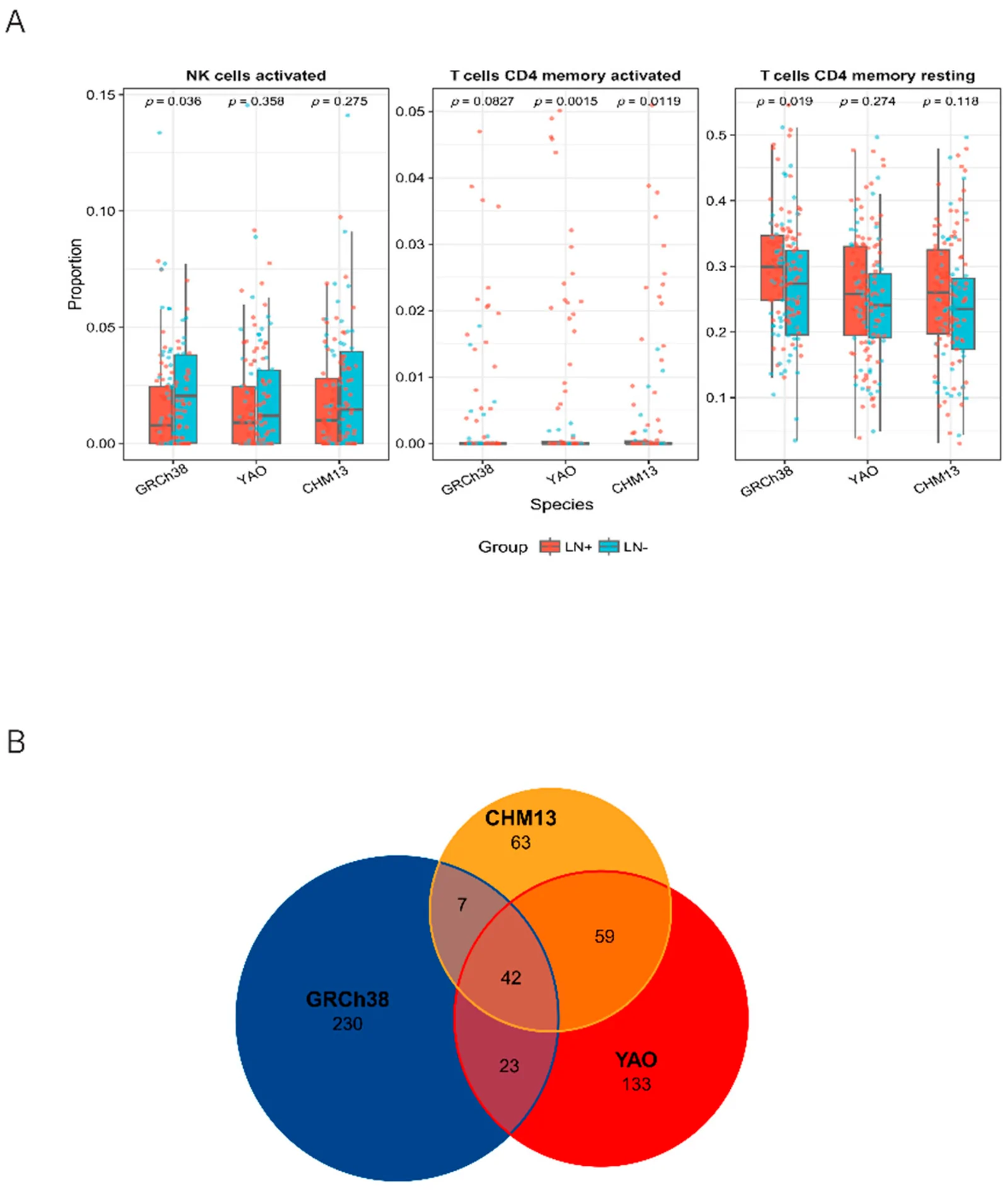
Influence of reference genome on immune microenvironment inference and fusion transcript detection. (**A**). Comparison of estimated infiltration levels for NK cells and memory T cells between LN+ and LN− conditions, as deconvoluted by CIBERSORT using expression profiles generated from GRCh38, YAO, and CHM13 reference genomes. (**B**). Venn diagram illustrating overlap and uniqueness of fusion transcripts identified by STAR-Fusion across GRCh38, YAO, and CHM13 reference genomes.

Given that reference genome choice influenced the cell type assignment for single cells, we next asked whether it similarly affects the inference of immune cell composition from bulk RNA-seq data, a common approach for characterizing the tumor microenvironment. Using CIBERSORT, we estimated difference in immune cell compositions between LN+ and LN− groups from expression profiles generated using YAO, CHM13 and GRCh38 (Figure 4A and Figure S8), where significant reference-specific patterns emerged for several immune subsets. For example, the inferred fractions of activated NK cells and CD4+ memory T cells (both activated and resting) showed different statistical significance patterns depending on the reference used (NK cell activated: GRCh38: *p* = 0.036, YAO: *p* = 0.358, CHM13: *p* = 0.275; CD4 memory T cell (activated): GRCh38: *p* = 0.0827, YAO: *p* = 0.0015, CHM13: *p* = 0.0119; CD4 memory T cell (resting): GRCh38: *p* = 0.019, YAO: *p* = 0.274, CHM13: *p* = 0.118). A global similarity assessment revealed that immune profiles derived from YAO and CHM13 were more correlated with each other (mean Pearson r = 0.849) than either was with GRCh38-derived profiles (r ~0.730) (Table S6).

### 3.6. Reference Genome Choice Influenced the Detection and Identification of Fusion Transcripts

Given that multi-gene families were particularly susceptible to mis-quantification due to paralogous sequence ambiguity, we hypothesized that reference genome completeness would similarly affect the detection of fusion transcripts, which relies on the precise alignment of chimeric reads spanning breakpoints.

Using STAR-Fusion to call fusion events from bulk RNA-seq data aligned to the three references, we observed substantial differences in both the number and composition of predicted fusions. GRCh38 produced the highest number of fusion calls (*n*= 302) collectively in all samples, followed by YAO (*n* = 257) and CHM13 (*n* = 171). However, the overlap among these callsets was limited (Figure 4B). Only 65 fusions were shared between GRCh38 and YAO and 49 between GRCh38 and CHM13. In contrast, the two T2T assemblies showed considerably greater concordance, sharing 101 fusion events. Although all predicted fusion events ultimately require independent validation, such as Iso-Seq [43], the markedly higher number of GRCh38-derived fusion calls relative to the T2T references suggests potential overestimation driven by mapping ambiguities in the incomplete GRCh38 assembly (Figure 4B). These findings further highlight the influence of reference genome selection on transcriptomic interpretation, particularly for analyses that depend on precise breakpoint-level alignment.

## 4. Discussion

In this study, we systematically evaluated how the selection of reference genomes affects various transcriptomic analyses, including quantification, differential expression analysis, predictive modeling, immune infiltration estimation, fusion transcript detection, and single-cell clustering. Overall, the major transcriptomic patterns were broadly preserved across the three reference genomes, while measurable reference-dependent effects were concentrated in a smaller subset of genes that were particularly sensitive to read assignment ambiguity. This overall concordance was evident in the re-mapping analysis, in which 80.09% ± 1.04% of reads uniquely assigned in YAO remained assigned to the same gene after realignment to GRCh38, as well as in differential expression analysis, where 65 DEGs were shared across all three references. These differences extend beyond read alignment or gene-level quantification and extend to disease group discrimination, immune cell component estimation, fusion transcript characteristics, and single-cell transcriptional state structure. Under the current dataset and analysis framework, analyses based on YAO and CHM13 generally show higher consistency in multiple downstream evaluation metrics, while the magnitude and direction of differences compared to analyses based on GRCh38 vary across different analysis tasks. It is notable that the overlap among candidate disease-related marker genes is higher than the differences observed in overall expression estimation. The reason for this phenomenon may be due to the small sample size. In conclusion, these findings suggest that the selection of reference genomes is an important yet often overlooked source of bias in cancer transcriptomics. Therefore, it should be considered in comparative studies, the interpretation of molecular phenotypes, and the development of transcriptome-based biomarkers.

Despite the reference-dependent differences in expression quantification, the three reference-based analyses retained a substantial proportion of shared disease-associated signals. For example, genes identified across the reference genomes included KAT6B [44] and CARTPT [45], which have previously been implicated in cancer-related transcriptional regulation and breast cancer biology, respectively. Genes associated with lipid metabolism and adipokine signaling, including ADIPOQ, ADIPOR1 [46], and PLIN1 [47], were also identified, consistent with the established involvement of metabolic regulation in luminal breast cancer. In addition, the expression pattern of JAK1 observed in our cohort was consistent with its previously reported association with breast cancer stage [48]. The recovery of these shared signals indicates that the major biological contrast between the two clinical groups was broadly preserved across reference genomes, even though the estimated expression levels and statistical significance of individual genes varied according to the reference used.

Given the relative nature of transcriptomic quantification, differences in reference genome choice can alter expression estimates and thereby affect feature selection and ranking. Under our analytical framework, classifier performance was broadly comparable across the three reference genomes, particularly in the independent validation cohort, and the modest numerical differences in AUC should not be interpreted as evidence that one reference is universally superior to another. Rather, YAO provides a population-matched T2T reference for the Chinese cohort, while reference genome choice itself can influence the composition and prioritization of candidate features. Notably, four features, KDM7A [39], SMARCD2 [40], RAD23B [41], and TINF2 [49], were selected exclusively in the YAO-derived model. Several of these genes have been implicated in breast cancer progression, stem cell maintenance, apoptosis, epithelial–mesenchymal transition, or breast cancer susceptibility, providing biological context for the YAO-specific feature set. The candidate marker sets largely overlapped between references, yet their priority rankings varied. Consistent with the comparable classifier performance, these findings suggest that the primary effect of reference choice in the present dataset lies in quantitative expression estimates and feature selection rather than a substantial improvement in overall predictive performance. In other disease contexts where such highly discrepant genes are more central, the impact of reference choice on model outcomes and biomarker discovery could be more pronounced, warranting further investigation in independent cohorts and functional experiments. Artificial intelligence (AI) is increasingly being applied to biomarker discovery and clinical research [50]. However, transcriptomics-based AI models rely on molecular features derived from upstream analyses, including reference genome alignment and gene expression quantification. Our findings suggest that reference genome choice may also influence AI-assisted biomarker discovery by affecting expression estimates and feature selection.

The observed improvements can be largely attributed to the ability of complete T2T assemblies to resolve the ambiguous mapping of reads derived from regions with high sequence similarity, a challenge that incomplete reference genomes cannot adequately handle. By providing a more comprehensive genomic representation, T2T assemblies enable better read placement and the more accurate quantification of paralogous genes [3,4]. While overall expression estimates are largely consistent between reference genomes, subtle read mapping discrepancies due to high sequence similarity may nevertheless propagate and affect downstream results. In particular, small expression shifts across multiple genes may be amplified during normalization [24], scaling, or group-wise pairing procedures, ultimately leading to substantial variability in differential expression, immune infiltration estimation, and other integrative analyses. For example, the fibroblast markers utilized, COL1A1, COL1A2, and COL3A1, all belong to the fibrillar collagen gene family and share high sequence homology [51]. Our findings demonstrate that incomplete reference genomes systematically convert this inherent biological sequence similarity into technical noise and misinterpretation. By providing a complete map of reference, T2T assemblies like CHM13 and YAO allow us to distinguish true biological variation in the expression of homologous genes (which may be population-specific) from mapping artifacts [52], thereby strengthening the foundation for any downstream inference.

In the analysis of fusion transcripts, we observed that each reference genome yielded a distinct set of reference-specific fusion events. These discrepancies are likely driven by differences in read mapping, as fusion detection is particularly sensitive to alignment uncertainty [32,53]. With second-generation short-read sequencing, individual genes are often supported by a limited number of uniquely assignable bases, increasing the likelihood of ambiguous or reference-dependent read assignment, especially in regions with high sequence similarity [54].

Therefore, our research also has some limitations. First, although our cohort included 148 Chinese breast cancer patients, the bulk RNA-seq samples were derived from a single population background and breast cancer subtype, which may limit the generalizability of our findings to other populations, tumor subtypes, and disease contexts. Moreover, the comparison of GRCh38, CHM13, and YAO does not allow the effects of reference completeness and population matching to be fully disentangled. Future studies incorporating additional population-specific T2T references and more genetically diverse cohorts will be needed to better distinguish these factors. Second, our analyses were primarily based on short-read RNA sequencing, which inherently limits resolution in highly homologous regions, complex splice junctions, and fusion breakpoints, and direct experimental validation using long-read transcriptome sequencing was not performed. Long-read sequencing technologies provide clear advantages in resolving full-length transcripts and complex fusion structures, thereby substantially reducing mapping-related biases [55,56]; however, their high cost and limited throughput currently restrict large-scale cohort applications. In this context, the use of population-matched reference genomes represents a cost-effective and scalable alternative to improve read assignment accuracy and mitigate short-read mapping bias without increasing experimental burden [57,58]. Third, although reference-dependent differences were observed in feature selection and predictive modeling, the relatively limited size of the independent validation cohort restricts conclusions regarding their clinical relevance. Larger multi-center cohorts will therefore be necessary to determine the reproducibility and clinical implications of these reference-dependent effects. These considerations position population-matched reference genomes as a practical solution for enhancing downstream transcriptomic analyses under realistic experimental constraints, while further validation across diverse populations and larger cohorts remains necessary.

## 5. Conclusions

Our results show that reference genome choice substantially influences transcriptomic analyses even under standardized analytical settings. T2T-based references improve mapping accuracy and quantification, while population-matched references further reduce bias and enable the detection of population-relevant transcriptomic features. The alteration in expression quantification extensively propagated to downstream DEG identification and biological interpretation. These findings underscore the importance of reference genome selection for accurate interpretation and biomarker discovery in transcriptomic studies.

## Data Availability

The datasets analyzed during the bulk RNA-seq study are available in the China National Center for Bioinformation/Beijing Institute of Genomics, Chinese Academy of Sciences (GSA-Human: HRA014762), and are publicly accessible at https://ngdc.cncb.ac.cn/gsa-human (accessed on 13 September 2026). The single cell RNA-seq datasets analyzed during this study are included in GEO and Gene Expression Omnibus under accession number GSE176078 in the published article at https://advanced.onlinelibrary.wiley.com/doi/full/10.1002/advs.202205395 (accessed on 13 September 2026). The YAO reference genome is available at https://ngdc.cncb.ac.cn/gwh under accession number GWHGEYC00000000.1 (accessed on 13 September 2026), and annotations were published on GitHub (https://github.com/KANGYUlab/T2T-YAO-resources (accessed on 13 September 2026)).

## Supplementary Materials

The following supporting information can be downloaded at: https://www.mdpi.com/article/10.3390/biomedicines14102296/s1, Figure S1. A comparative analysis of transcript identities between the YAO and GRCh38 reference genomes. Figure S2. Comparative assessment of basic alignment performance between YAO and GRCh38 reference genomes. A. Mapping ratio across samples. B. Mismatch ratio across samples. C. Multi-mapping ratio across samples. Figure S3. GO enrichment analyses were performed for the 8432 genes exhibiting more than a ten-fold difference in expression between YAO and GRCh38 across the analyzed samples. Figure S4. A. The gene structure and copy number of the KCNJ12 and KCNJ18 genes on YAO and GRCh38. B. The unique alignment reads of the representative KCNJ18 gene on YAO, realigned in GRCh38. C. ClustalX was used to display the similarity of the exons of copy 2 of the KCNJ12 and KCNJ18 genes. Figure S5. A. A comparison of the genetic structure of NPIPA2 in YAO, GRCh38, and NPIPA1 in GRCh38. B. Distribution of 17 read pairs that uniquely mapped to NPIPA2 on YAO and were realigned to GRCh38. C. ClustalX alignment showing the sequence similarity among the last exon of NPIPA2 in YAO, exons 8 and 10 of NPIPA1 in GRCh38, and exons 8 and 28 of NPIPA2 in GRCh38. Figure S6. Performance evaluation of random forest models built from DEG sets identified by VTwins across reference genomes. A. Three reference genomes were used to select 20 DEGs, and B. their AUC values were calculated in the random forest model in both the training set and the validation set. Figure S7. The expression levels of canonical fibroblast markers COL1A1, COL3A1, and ACTA2 in the fibroblast cluster identified using the GRCh38, CHM13, and YAO reference genomes. COL2A1 was excluded from this analysis as it was filtered out during the HVG selection step in both the CHM13 and YAO analyses. Figure S8. A comparative analysis of infiltration levels for the remaining 19 immune cell types, as deconvoluted by CIBERSORT across the three reference genomes. Table S1. A summary of clinical characteristics and sequencing data for the patient cohort. Table S2. The importance (MeanDecreaseGini) of using DEGs for random forest modeling in GRCh38 and CHM13. Table S3. Predictive performance of random forest classifiers built using varying numbers of top VTwins-derived DEGs. Table S4. Assessment of clustering consistency based on pairwise comparisons among GRCh38, YAO, and CHM13 reference genomes. Table S5. Identification of cluster-specific DEGs through analysis of three reference genomes. Table S6. Results of Pearson correlation analysis for cell evaluation across all samples within reference genome.

## Abbreviations

The following abbreviations are used in this manuscript:

T2T	telomere-to-telomere
LN	lymph node metastasis
*p*.adjust	adjusted *p*-value
log2FC	log2 fold change
ROC	receiver operating characteristic
AUC	area under the curve
HVGs	highly variable genes
PCA	principal component analysis
UMAP	uniform manifold approximation and projection
NK	natural killer
ARI	adjusted Rand index
NMI	normalized mutual information
CTAs	cancer/testis antigens
FAM	family with sequence similarity
DEGs	differentially expressed genes
RF	random forest
