# Supplementary material for: Reference Genome Choice Shapes RNA-Seq Quantification and Downstream Interpretation in Chinese Breast Cancer Patients
Source: Biomedicines. 2026 Oct 9;14(10):2296. doi: 10.3390/biomedicines14102296 (PMC13701484; doi:10.3390/biomedicines14102296)
Supplement: Supplementary file 1 [file biomedicines-14-02296-s001.zip › biomedicines-4594701-supplementary/supplemet files/SF—new.pdf]

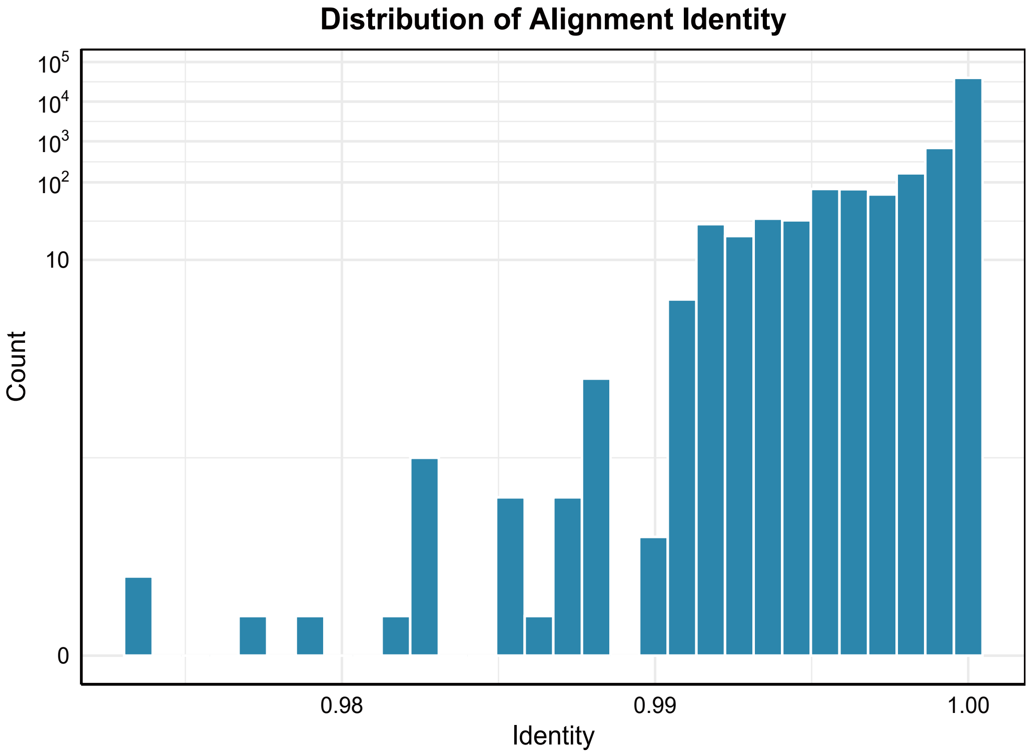

Figure S1. A comparative analysis of transcript identities between the YAO and GRCh38 reference genomes.

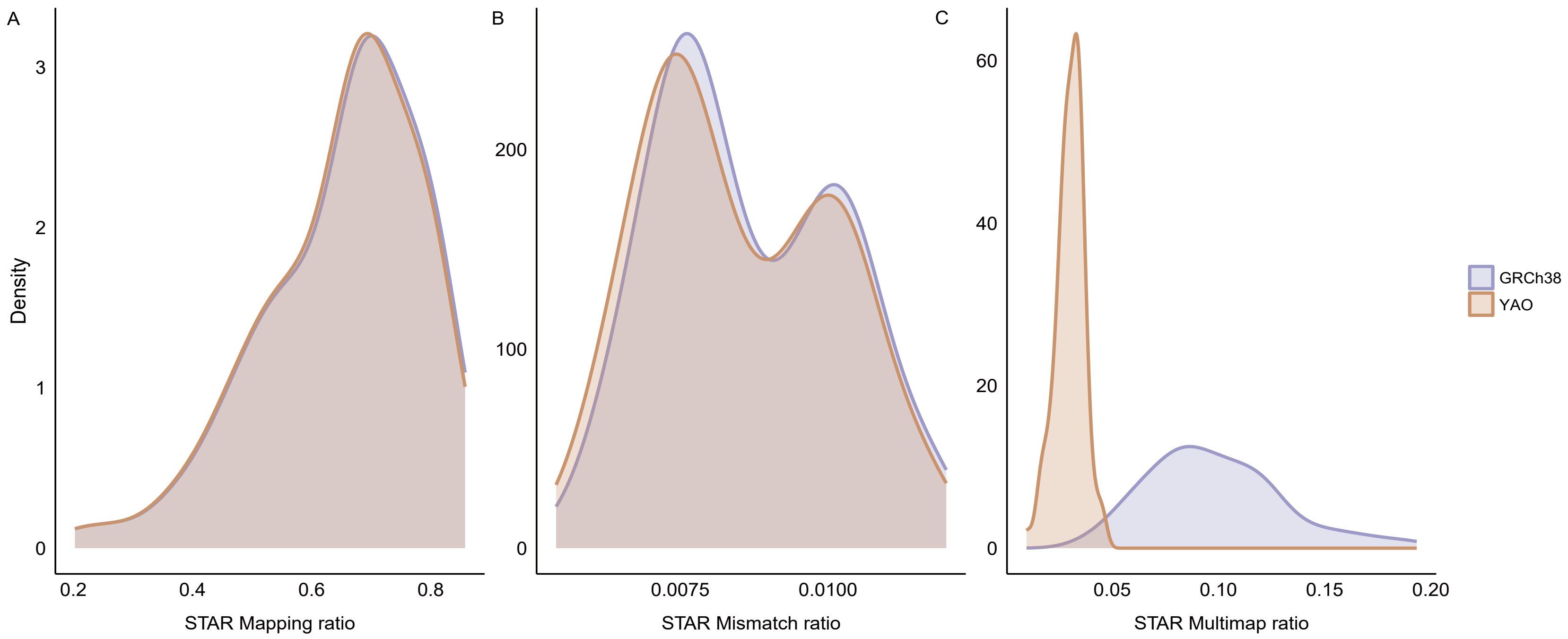

Figure S2. Comparative assessment of basic alignment performance between YAO and GRCh38 reference genomes. A. Mapping ratio across samples. B. Mismatch ratio across samples. C. Multi-mapping ratio across samples.

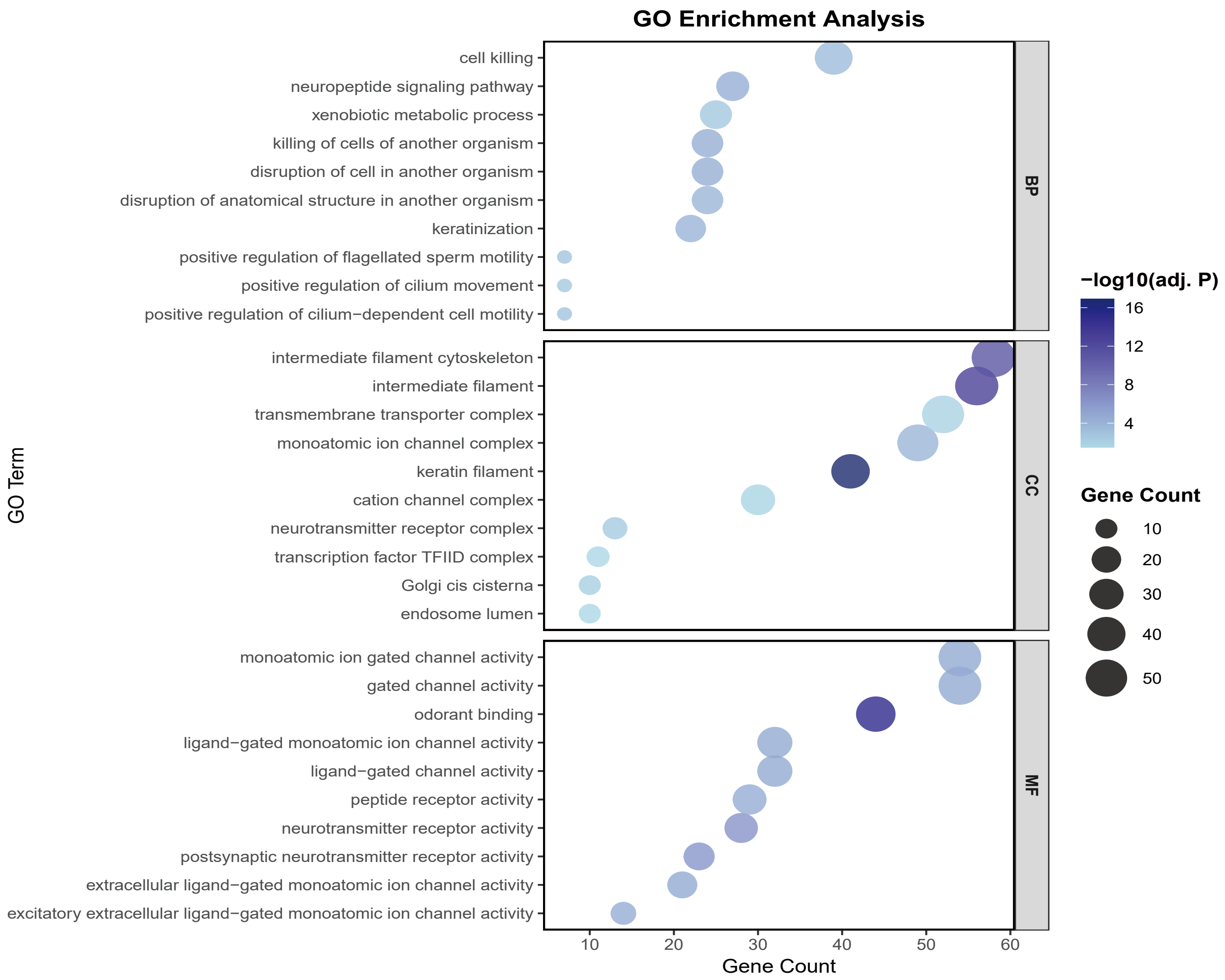

Figure S3. GO enrichment analyses were performed for the 8432 genes exhibiting more than a ten-fold difference in expression between YAO and GRCh38 across the analyzed samples.

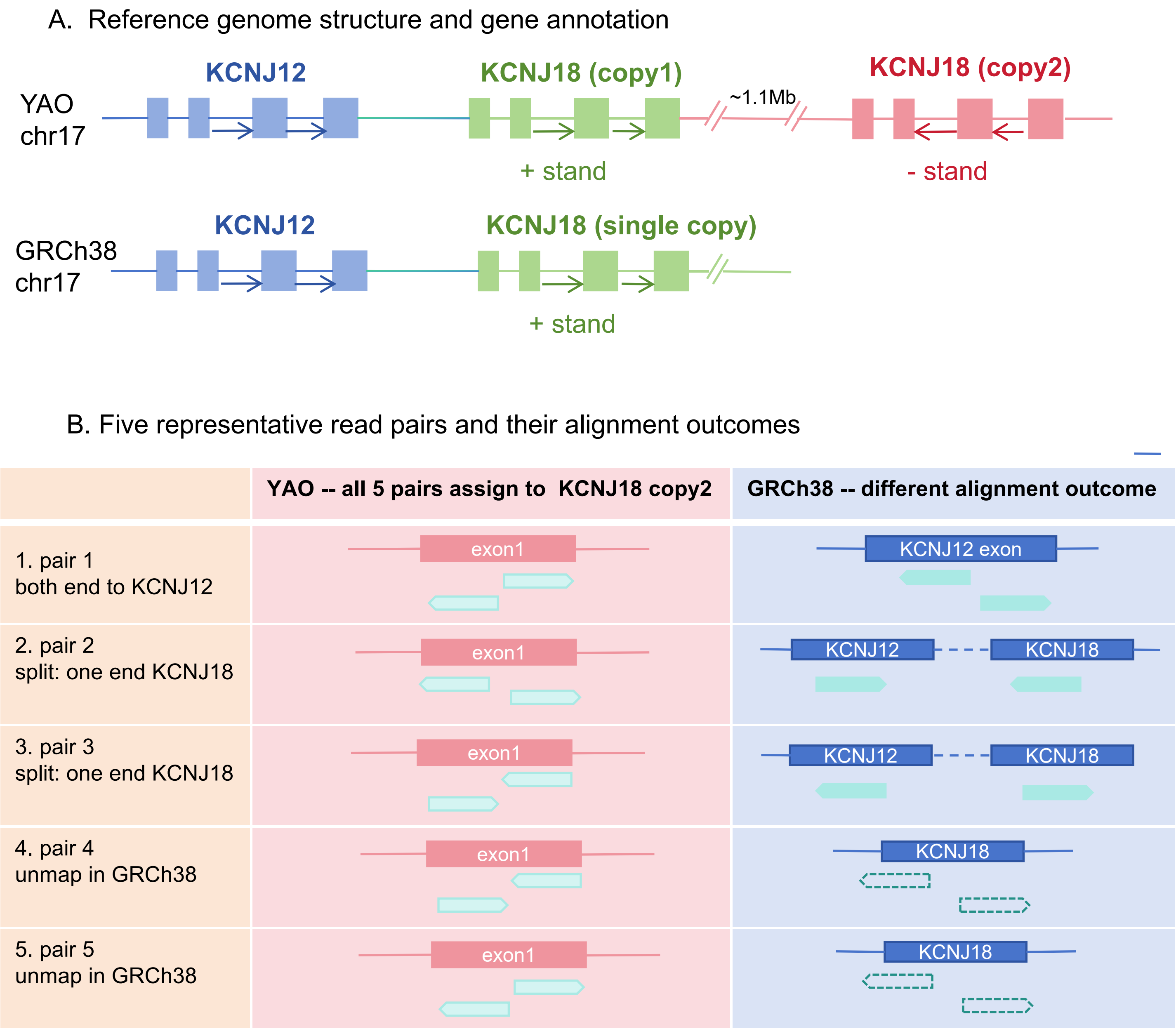
A. Reference genome structure and gene annotation
KCNJ12
KCNJ18 (copy1)
KCNJ18 (copy2)
YAO chr17
~1.1Mb
+ stand
- stand
KCNJ12
KCNJ18 (single copy)
GRCh38 chr17
+ stand
B. Five representative read pairs and their alignment outcomes
YAO -- all 5 pairs assign to KCNJ18 copy2
GRCh38 -- different alignment outcome
1. pair 1
both end to KCNJ12
exon1
KCNJ12 exon
2. pair 2
split: one end KCNJ18
exon1
KCNJ12
KCNJ18
3. pair 3
split: one end KCNJ18
exon1
KCNJ12
KCNJ18
4. pair 4
unmap in GRCh38
exon1
KCNJ18
5. pair 5
unmap in GRCh38
exon1
KCNJ18

C. Identity between KCNJ12 and KCNJ18 copy2 exon in YAO and GRCh38

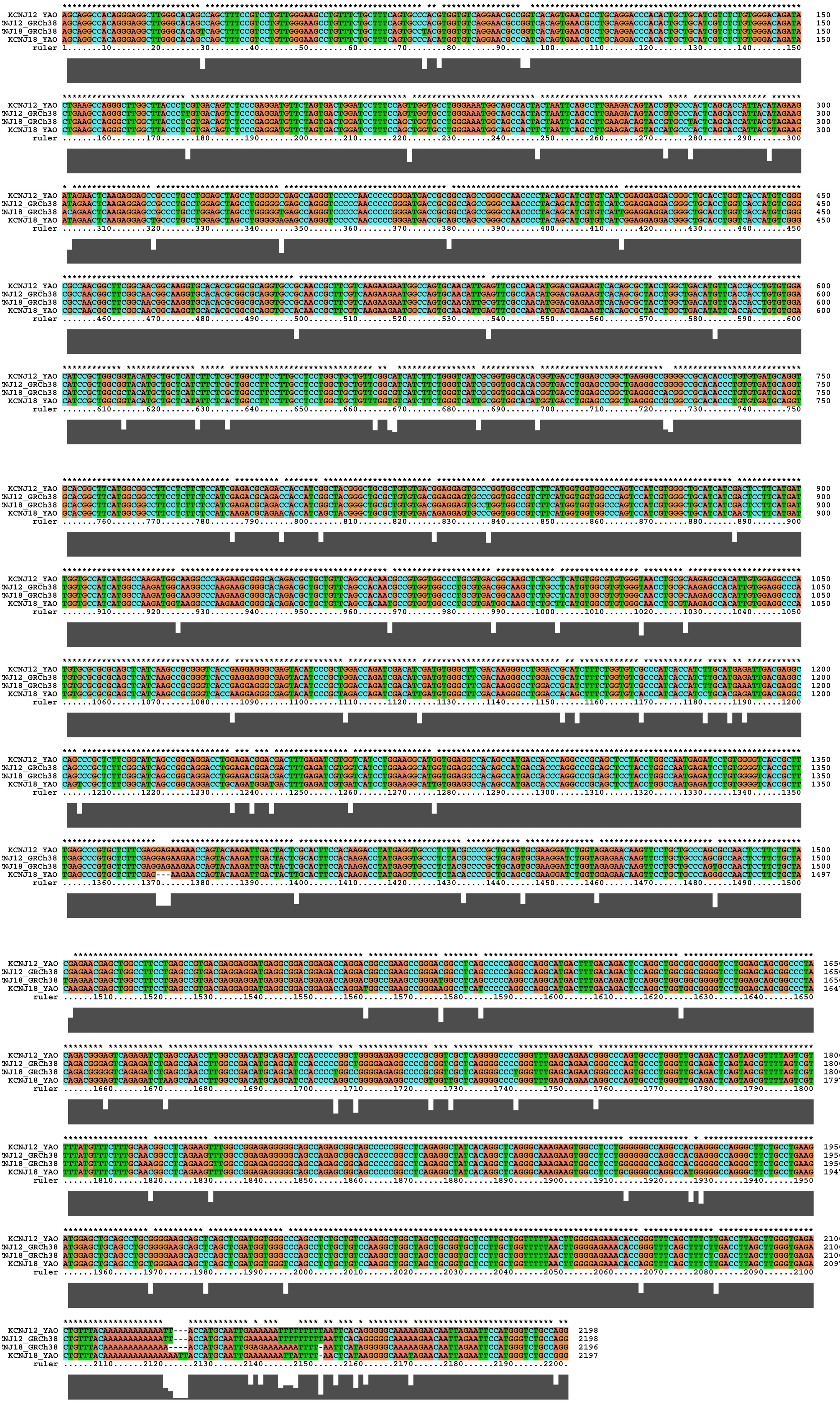

Figure S4. A. The gene structure and copy number of the KCNJ12 and KCNJ18 genes on YAO and GRCh38. B. The unique alignment reads of the representative KCNJ18 gene on YAO, realigned in GRCh38. C. ClustalX was used to display the similarity of the exons of copy 2 of the KCNJ12 and KCNJ18 genes.

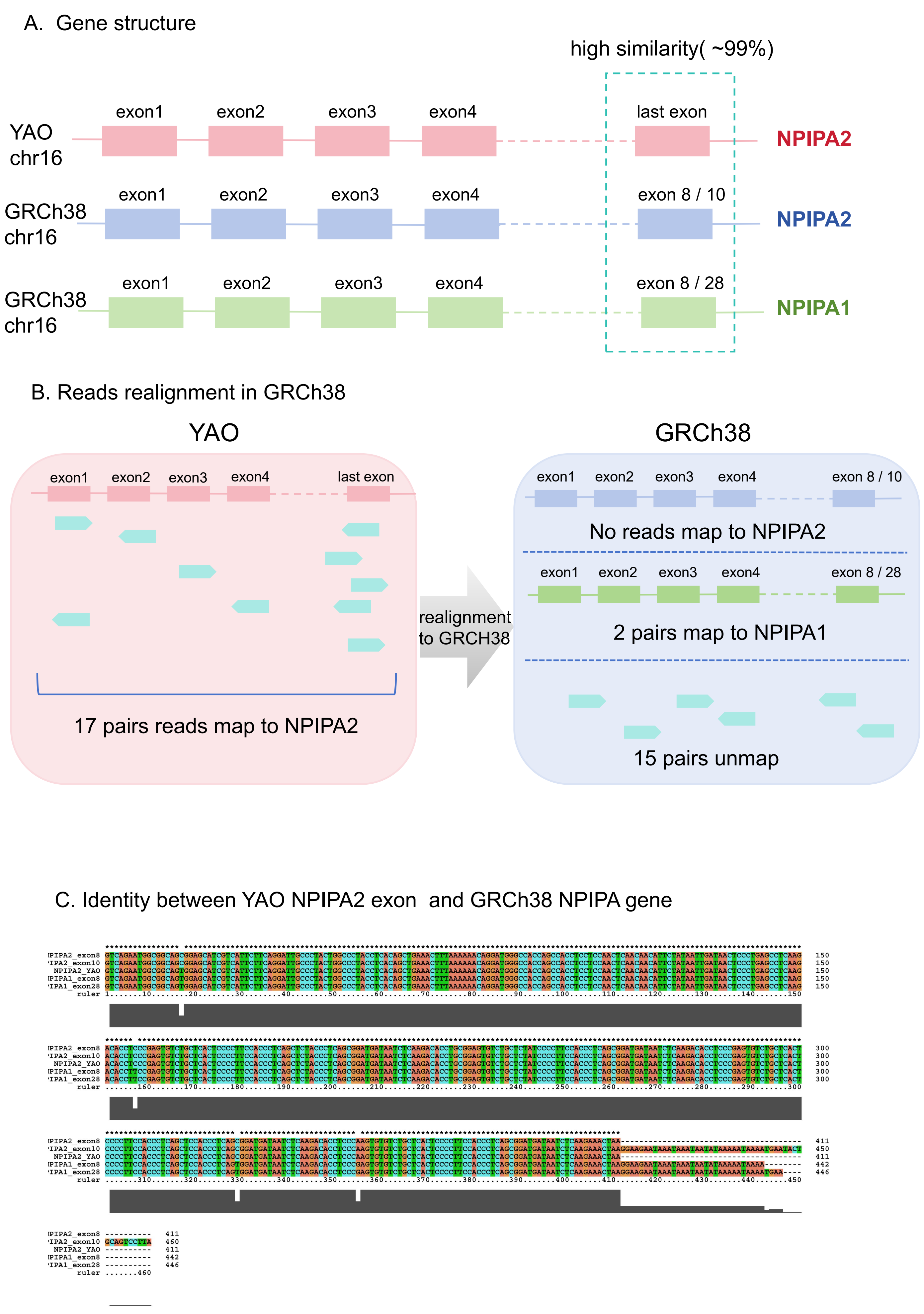

Figure S5. A. A comparison of the genetic structure of NPIPA2 in YAO, GRCh38, and NPIPA1 in GRCh38. B. Distribution of 17 read pairs that uniquely mapped to NPIPA2 on YAO and were realigned to GRCh38. C. ClustalX alignment showing the sequence similarity among the last exon of NPIPA2 in YAO, exons 8 and 10 of NPIPA1 in GRCh38, and exons 8 and 28 of NPIPA2 in GRCh38.

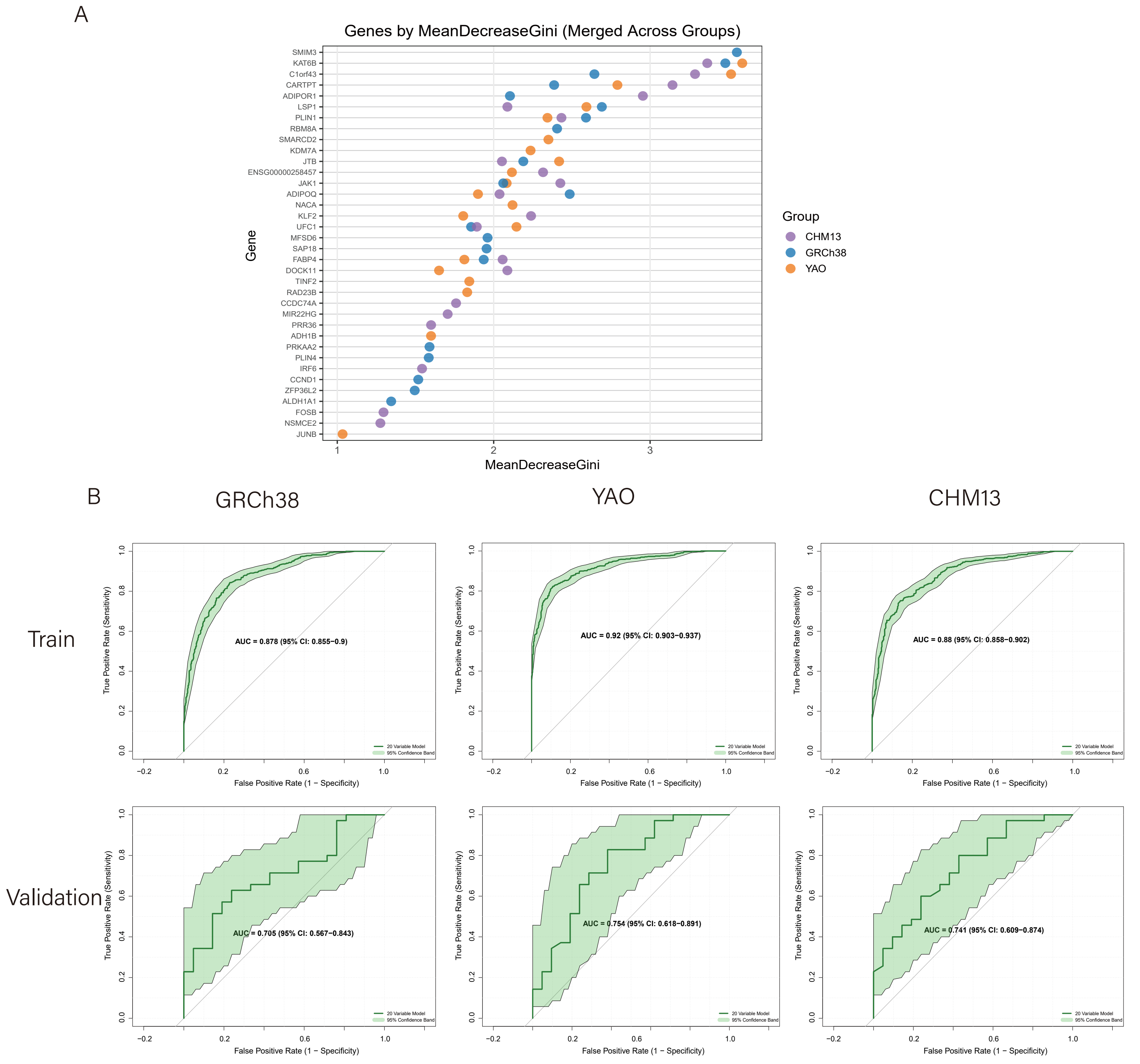

Figure S6. Performance evaluation of random forest models built from DEG sets identified by VTwins across reference genomes. A. Three reference genomes were used to select 20 DEGs, and B. their AUC values were calculated in the random forest model in both the training set and the validation set.

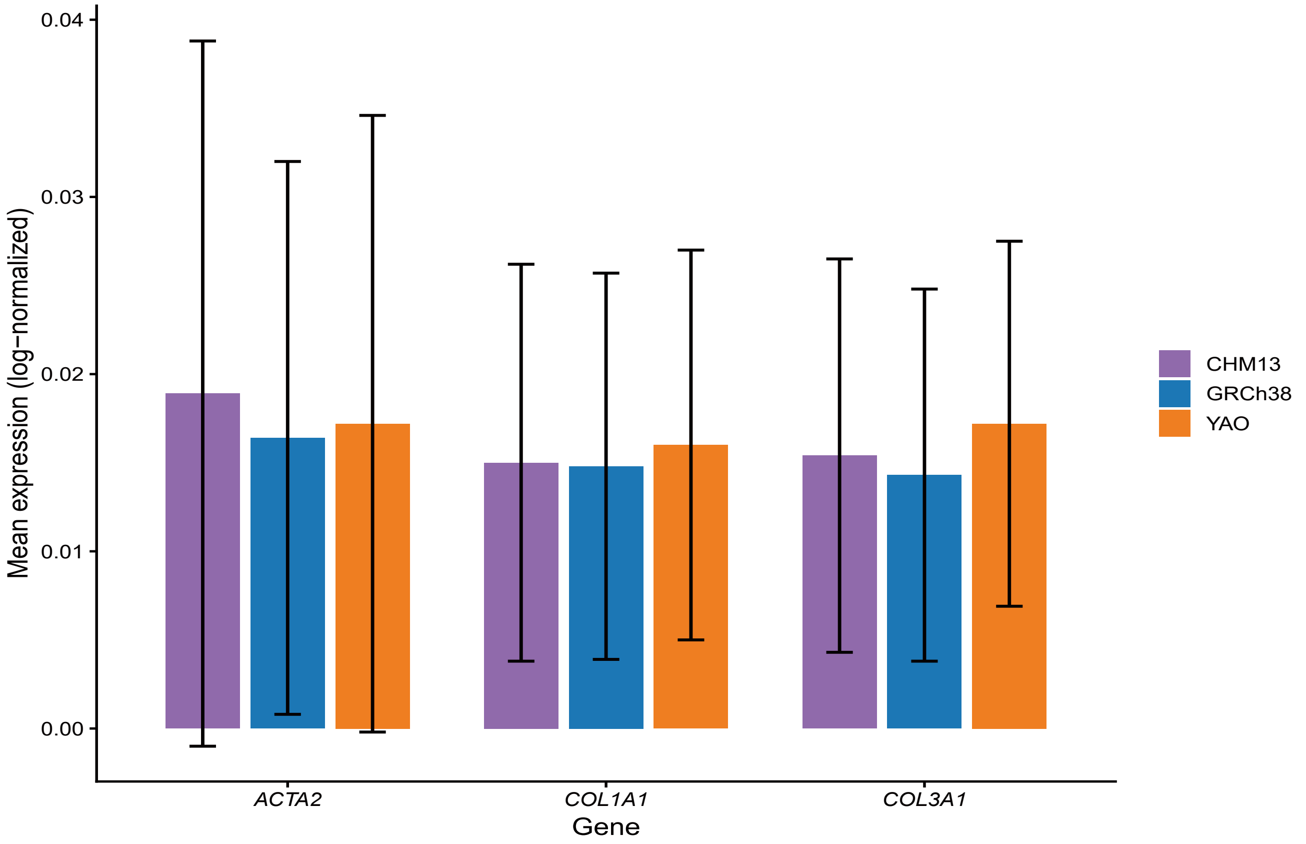

Figure S7.The expression levels of canonical fibroblast markers COL1A1, COL3A1, and ACTA2 in the fibroblast cluster identified using the GRCh38, CHM13, and YAO reference genomes. COL2A1 was excluded from this analysis as it was filtered out during the HVG selection step in both the CHM13 and YAO analyses.

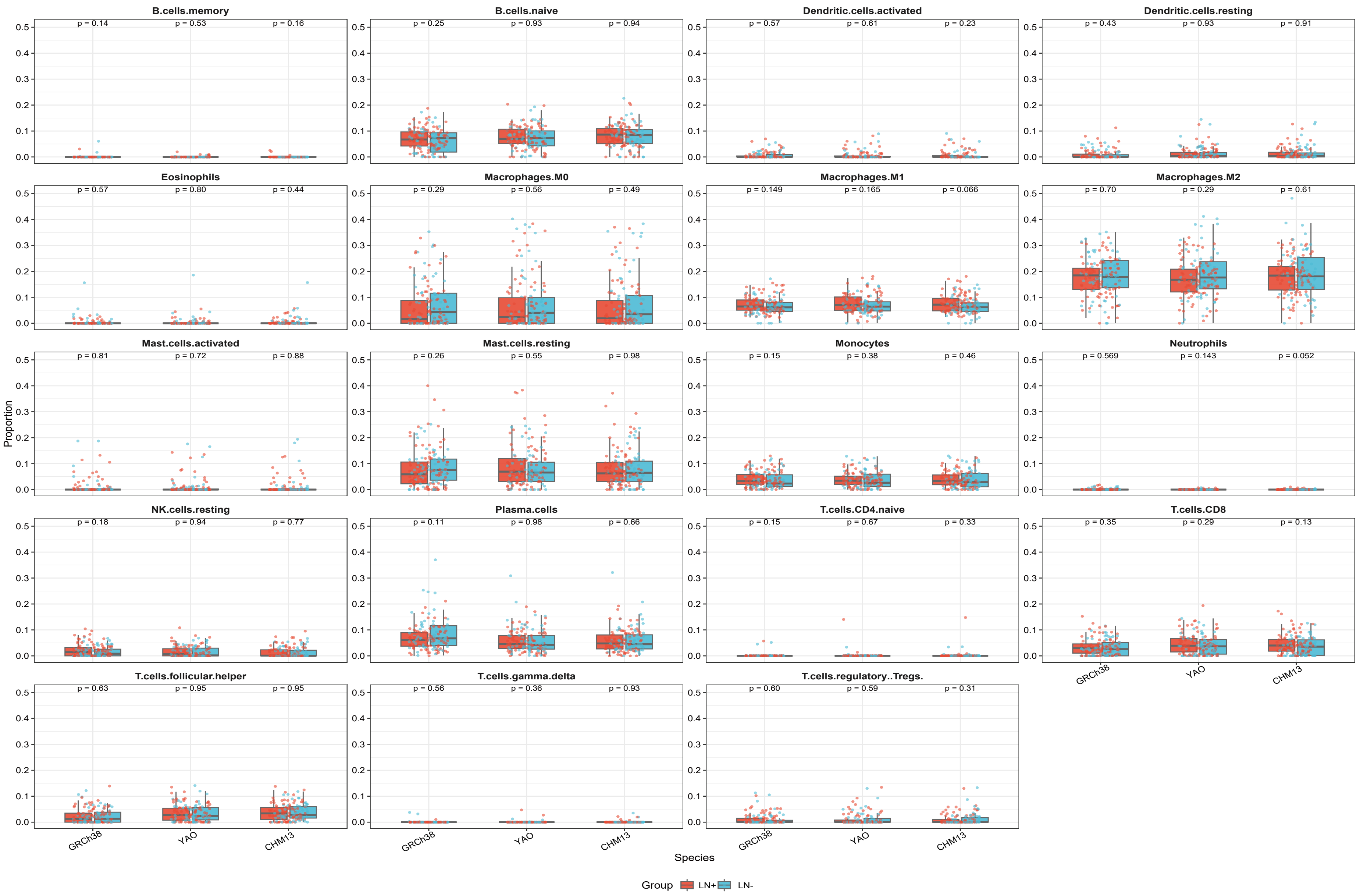

Figure S8. A comparative analysis of infiltration levels for the remaining 19 immune cell types, as deconvoluted by CIBERSORT across the three reference genomes.
